# ΔNp63*α* modulates histone methyl transferase SETDB1 to transcriptionally repress target genes in cancers

**DOI:** 10.1038/cddiscovery.2016.15

**Published:** 2016-02-29

**Authors:** C Regina, M Compagnone, A Peschiaroli, AM Lena, G Melino, E Candi

**Affiliations:** 1 Department of Experimental Medicine and Surgery , University of Rome ‘Tor Vergata’, Via Montpellier 1, Rome 00133, Italy; 2 Institute of Cell Biology and Neurobiology (IBCN), CNR, Rome, Italy; 3 IDI-IRCCS, Biochemistry Laboratory, Via Monti di Creta 104, Rome 00166, Italy

ΔNp63*α* is primarily expressed in the epithelial tissue, including the mammary gland and epidermis, where it is indispensable to maintain the high proliferative potential of somatic stem cells.^1^ Although mutations of p63 are extremely rare in human cancers, several tumors, including primary head and neck squamous cell carcinomas (HNSCCs), squamous cell epithelial lung malignancies, non-small-cell lung cancers and basal-like subtypes of breast cancer,^2–6^ often display elevated levels of ΔNp63, which is associated with poor prognosis. However, the mechanism of action of ΔNp63*α* in tumors remains mostly unknown. ΔNp63 isoforms, the amino-deleted isoforms encoded by TP63, lack the N-terminal transactivation (TA) domain, but are still able to transcriptionally regulate a distinct subset of genes due to the presence of a second TA domain (TA2). Thus, ΔNp63*α* has been shown to function both as a transcriptional activator and as a transcriptional repressor. Although the ΔNp63 transcriptional profile has been extensively characterized in normal epithelial cells and in cancer cell lines, little is known about how ΔNp63*α* directly transactivates genes, and which co-activators are required at enhancer and promoter sites. Furthermore, detailed information precisely mapping the TA2 domain is still missing. In contrast, much more information is available on the mechanisms of ΔNp63*α*-mediated transcriptional repression. ΔNp63*α* represses transcription by directly antagonizing p53 family members or by modulating the chromatin landscape near target genes (Figure 1). In the past several years, one prevalent hypothesis in the literature has been that ΔNp63*α* represses TAp73/p53 target genes simply by acting as dominant-negative to prevent TAp73/p53 occupancy at the shared DNA responsive elements (Figure 1a). For example, p63 knockdown in HNSCC cell lines results in TAp73-dependent apoptosis via PUMA and NOXA upregulation. In this system, ΔNp63*α* forms hetero-tetramers with TAp73, preventing the binding of TAp73 to PUMA enhancers.^7^ Although this notion is still valid, it did not explain several results obtained in HNSCC^2^ and in other cancer types suggesting that alternative TAp73/p53-independent mechanisms, employed by ΔNp63*α*, are engaged. Indeed, in keratinocytes and in HNSCC cell lines, ΔNp63*α* physically interacts with the histone deacetylases HDAC1 and HDAC2, and recruits these enzymes to p63 and p53 enhancer sites, thus mediating histone H3 and H4 deacetylation and consequent transcriptional inhibition (Figure 1b).^8^ Another ΔNp63*α*-dependent mechanism of repression is the recruitment of the SRCAP chromatin remodelling complex, via a physical interaction with the SAMD9L subunit.^2^ SRCAP complex is involved in H2A/H2A.Z exchange, mediating H2A.Z deposition near p63 response elements, thus creating a chromatin environment that is in a repressed conformation; this has been demonstrated in keratinocytes, lung SCC and HNSCC cell lines (Figure 1c).

Recently, using a yeast two-hybrid assay, Regina *et al.* ^9^ showed that ΔNp63*α* interacts with SETDB1, a histone lysine methyl transferase (HMT) that is important in epigenetic regulation (Figure 1d). SETDB1 belongs to the SET (Suppression of variegation, Enhancer of zeste, Trithorax)-domain containing enzymes. HMTs catalyze the transfer of one to three methyl groups from *S*-adenosyl-methionine to specific lysine residues on histone proteins.^10^ Depending on the site and degree of methylation, this modification can have various effects, including regulation of chromatin organization and gene transcription. Among the different HMTs, SETDB1 has been of increasing interest due to its involvement in melanoma, where it is located in a recurrently amplified chromosome fragment.^11^ SETDB1 amplification has been also described in lung tumors.^12^ Regina *et al*.^9^ demonstrated that SETB1 is also overexpressed in different breast cancer cell lines and in primary tumors. Knockdown of SETDB1 resulted in growth-inhibitory effects. The authors also identified a list of 30 genes possibly repressed by ΔNp63 in a SETDB1-dependent manner, some of which correlated with the survival of breast cancer patients, suggesting that the ΔNp63*α*−SETDB1 interaction has a relevant and functional role in breast tumorigenesis.

These findings indicate a third mechanism through which ΔNp63*α* represses transcription, demonstrating that ΔNp63*α* uses different partners in a combinatorial fashion and in a cell-type-specific manner. Understanding mechanistically how ΔNp63*α* recruits chromatin remodelers, and identifying repressed target genes in different cells and cancer types, could be important in the future to modulate senescence/proliferation in epithelial cells and to block rapid cancer expansion.

## Figures and Tables

**Figure 1:**
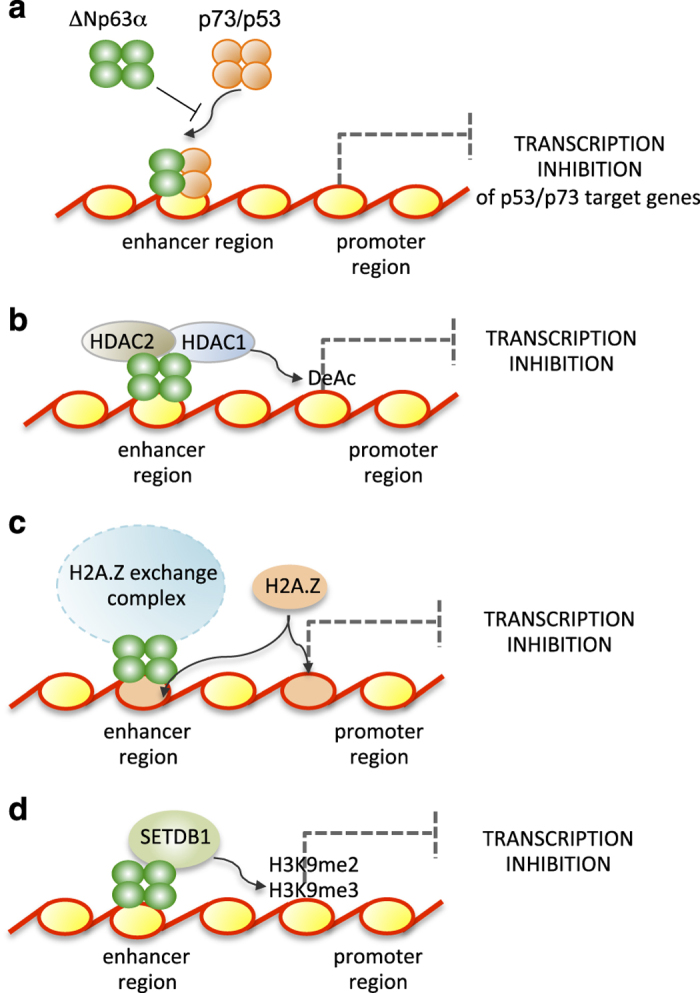
Schematic view of different mechanisms of ΔNp63*α*-mediated inhibition in different cancer types. (**a**) ΔNp63*α*, by direct interaction with p53-like responsive elements and/or by forming mixed inactive tetramers, inhibits the transcription of TAp73/p53 target genes, acting in a dominant-negative fashion. This mechanism has been demonstrated in keratinocytes and HNSCCs.^7^ (**b**) ΔNp63*α*, by physical interaction with the histone deacetylases HDAC1 and HDAC2, recruits these enzymes to chromatin, resulting in deacetylation of histone H4 and consequent transcription inhibition. This has been shown in JHU-029 SCC cell line.^8^ (**c**) ΔNp63*α* recruits components of the H2A.Z exchange complex to facilitate H2A.Z incorporation to repress transcription. This mechanism has been observed in the lung SCC cell line H226.^2^ (**d**) ΔNp63*α*, by physical interaction with the histone lysine methyl transferases SETDB1, may repress transcription of target genes^9^ by SETDB1 deposition of histone H3 lysine 9 dimethylation and of histone H3 lysine 9 trimethylation marks. This mechanism has been observed in breast cancer cell lines.^9^
